# Conventional methanotrophs are responsible for atmospheric methane oxidation in paddy soils

**DOI:** 10.1038/ncomms11728

**Published:** 2016-06-01

**Authors:** Yuanfeng Cai, Yan Zheng, Paul L. E. Bodelier, Ralf Conrad, Zhongjun Jia

**Affiliations:** 1 State Key Laboratory of Soil and Sustainable Agriculture, Institute of Soil Science, Chinese Academy of Sciences, Nanjing, Jiangsu Province 210008, China; 2 School of Food and Bioengineering, Zhengzhou University of Light Industry, Zhengzhou, Henan Province 450002, China; 3 Netherlands Institute of Ecology (NIOO-KNAW), Droevendaalsesteeg 10, 6708PB Wageningen, The Netherlands; 4 Max Planck Institute for Terrestrial Microbiology, Karl-von-Frisch-Strasse 10, D-35043 Marburg, Germany

## Abstract

Soils serve as the biological sink of the potent greenhouse gas methane with exceptionally low concentrations of ∼1.84 p.p.m.v. in the atmosphere. The as-yet-uncultivated methane-consuming bacteria have long been proposed to be responsible for this ‘high-affinity' methane oxidation (HAMO). Here we show an emerging HAMO activity arising from conventional methanotrophs in paddy soil. HAMO activity was quickly induced during the low-affinity oxidation of high-concentration methane. Activity was lost gradually over 2 weeks, but could be repeatedly regained by flush-feeding the soil with elevated methane. The induction of HAMO activity occurred only after the rapid growth of methanotrophic populations, and a metatranscriptome-wide association study suggests that the concurrent high- and low-affinity methane oxidation was catalysed by known methanotrophs rather than by the proposed novel atmospheric methane oxidizers. These results provide evidence of atmospheric methane uptake in periodically drained ecosystems that are typically considered to be a source of atmospheric methane.

## Abstract (web summary)

Atmospheric methane may be consumed by microorganisms in soil, but the mechanisms behind high-affinity methane oxidization remain poorly understood. Here, Jia *et al*. show that known methanotrophic bacteria are responsible for atmospheric methane uptake in periodically drained wetland ecosystems.

## Graphical abstract

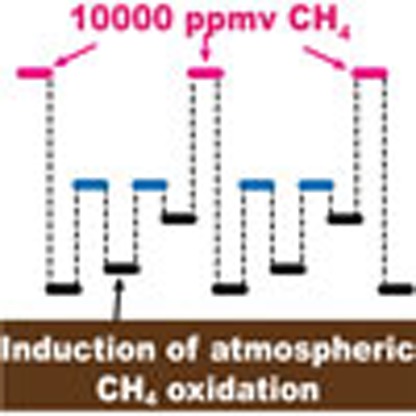

Methane (CH_4_) is the second most important atmospheric greenhouse gas after CO_2_ and is believed to account for 17% of global warming^1^. Atmospheric methane is consumed by microorganisms in aerobic upland soils that are predominantly exposed to near- or sub-atmospheric methane concentrations at the exceptionally low level of ∼1.84 p.p.m.v. (ref. 2). Theoretical calculations of soil methane consumption dynamics indicate that atmospheric methane does not supply enough maintenance energy for any known methanotrophs to survive^3^. Existing evidence regarding responsible microbes that possess high-affinity methane oxidation (HAMO) enzymes points to as-yet-uncultured methanotrophic lineages. These lineages are comprises upland soil cluster alpha (USCα) and upland soil cluster gamma (USCγ), which are frequently detected in aerobic upland soils, such as those in forest and grassland regions, but not in wetland soils^4^,^5^,^6^.

Wetland ecosystems typically harbour high concentrations of methane, which are generated as the end product of the anaerobic degradation of organic matter. In this setting, the well-known ‘low-affinity' methanotrophs, which contain the type I (*Gammaproteobacteria*) and type II (*Alphaproteobacteria*) subgroups, catalyse methane oxidation at the aerobic–anaerobic interfaces. These interfaces include the oxygenated surface soil layers and the area around the oxygen-releasing roots of wetland plants^7^. It is estimated that >80% of endogenous high concentrations of methane produced in anaerobic compartments are consumed by methanotrophs before escaping to the atmosphere^8^,^9^. Intriguingly, during the drought season, field measurements have suggested that the HAMO activity occurred in a swamp due to the direct consumption of atmospheric methane at 1.84 p.p.m.v., whereas the swamp soils function as a net source of methane under waterlogged conditions^10^. This shift between methane source and sink has also been found in many other types of wetlands with fluctuating water levels, such as peatlands^11^, tidal freshwater wetlands^12^ and acidic wetlands^13^. However, the mechanisms of atmospheric methane oxidation remain elusive in these periodically draining wetlands.

Rice paddies can serve as a model system for periodically draining wetland ecosystems because the practice of agricultural rotation is commonly used with waterlogged rice cultivation and upland wheat in Asia. In addition, the mid-season drainage of a rice field can lead to significant fluctuations in the soil methane concentrations owing to the varying water content. Thus, we used paddy soil as a model system to mimic the fluctuating availability of methane in periodically draining wetland soils by exposing it to various methane mixing ratios. Results showed that the HAMO activity for 1.84 p.p.m.v. methane uptake emerged only after soil oxidation of methane at 10,000 p.p.m.v. and ceased after 2 weeks, but could be regained after re-exposing the soil to 10,000 p.p.m.v. methane. Feeding the soil with 10,000 p.p.m.v. methane for 10 days on a daily basis led to a greater endurance of HAMO activity, although the growth of methanotrophic populations was progressively constrained. A metatranscriptome-wide association study (MTWAS) revealed the strong transcriptional activity of key enzymes of conventional methane oxidizers, indicating that various intracellular polymers could serve as reducing equivalents in support of soil HAMO activity. Our results demonstrate that conventional methanotrophic bacteria are responsible for atmospheric methane oxidation in periodically draining wetlands, which are typically considered to be a source of atmospheric methane.

## Results and Discussion

### Emergence and resilience of soil HAMO activity

**Figure 1:**
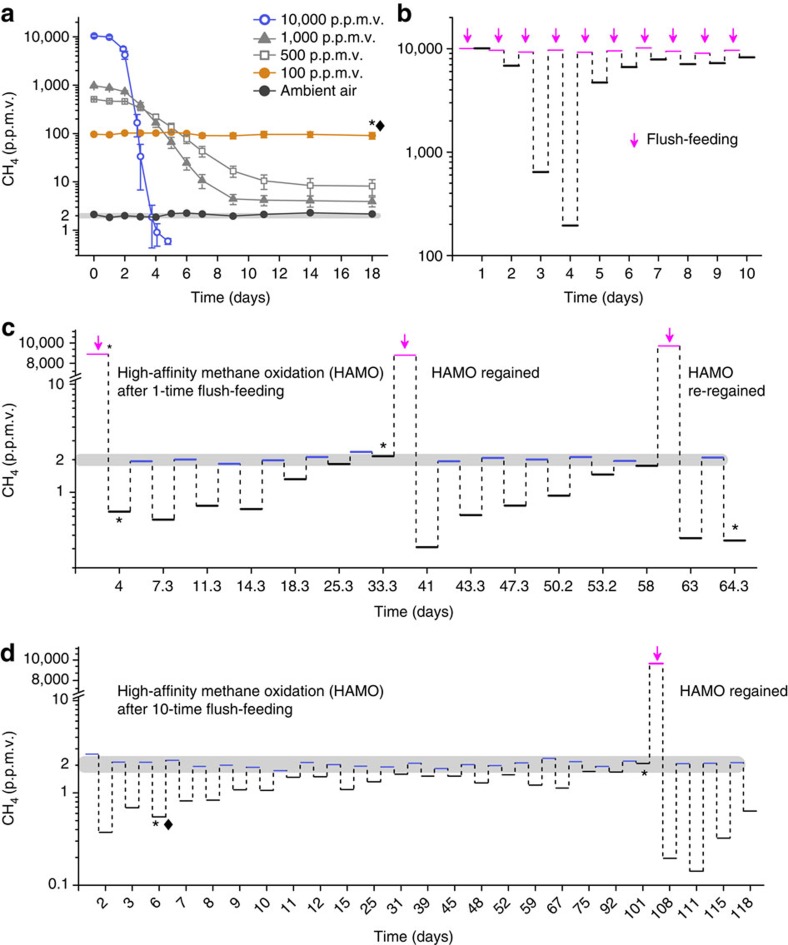
Emergence and resilience of high-affinity methane oxidation activity in paddy soil. (**a**) The consumption dynamics of methane under different initial mixing ratios. High-affinity methane oxidation (HAMO) activity occurred only in soil amended with 10,000 p.p.m.v. The error bars represent 2 s.d. of measurements taken from triplicate microcosms. (**b**) Methane consumption changes in 10-time flush-feeding microcosms with 10,000 p.p.m.v. methane. The headspace methane was replaced every day to maintain 10,000 p.p.m.v. methane. Red bar: initial methane mixing ratio after each replacement; black bar: methane mixing ratio after 1 day of incubation. (**c**,**d**) Gradual losses and regain of HAMO activity are shown after 1-time (**c**) and 10-time (**d**) flush-feeding. The light grey region represents the range of methane mixing ratios in the atmosphere during the experiment, and the black bar indicates the concentration of methane left in soil microcosms after several days' incubation. The black diamonds in **a**–**d** indicate the soil samples with and without HAMO activity that were used for the metatranscriptomic analysis, respectively. The black asterisks indicate the soil samples used for quantitative PCR analysis of the *pmoA*2 genes encoding a novel particulate methane monooxygenase with a high affinity for methane and for high-throughput Illumina sequencing of 16S rRNA genes and transcripts. All data shown in **b**–**d** represent the average values derived from triplicate microcosms.

We investigated methane oxidation dynamics by incubating paddy soil under aerobic conditions with methane at five different mixing ratios, including ∼2 p.p.m.v. in the ambient condition and 100, 500, 1,000 and 10,000 p.p.m.v. for the elevated concentrations. No methane oxidation occurred in the soil when exposed to ambient air and 100 p.p.m.v. methane over the 18-day incubation period (Fig. 1a). However, in the 10,000 p.p.m.v. methane-amended microcosms, the methane concentration dropped rapidly to ∼2 p.p.m.v. within 3 days and further decreased to as low as 0.6 p.p.m.v. (Fig. 1a), suggesting the emergence of HAMO activity in this soil. Intriguingly, in the 500 and 1,000 p.p.m.v. methane-amended microcosms, HAMO activity was not induced, although the headspace methane concentration decreased to ∼10 and 4 p.p.m.v., respectively. These results suggest that a critical level of methane turnover might be required for the induction of HAMO activity in paddy soils.

To verify the phenomenon of emerging of HAMO activity and to assess its dependence on methane turnover, we incubated the soil again with a single exposure to 10,000 p.p.m.v. methane (1-time flush-feeding, 1-TF) or incubated it with 10,000 p.p.m.v. methane for 10 days (10-time flush-feeding, 10-TF) by renewing the headspace gas once a day (Fig. 1b). During the 10-day incubation period, a marked increase of the methane consumption of the soil was observed after 3–4 days of incubation. However, subsequent replenishment of the 10,000 p.p.m.v. methane led to a steady decrease in the methane oxidation, pointing towards possible nutrient deprivation given the excessive supply of methane-derived carbon (Fig. 1b). We then refreshed these 1-TF and 10-TF soil groups with ambient air to determine their HAMO activity. It is noteworthy that the HAMO activity was not higher in 10-TF than the 1-TF soils, despite the fact that a threefold higher amount of methane was cumulatively oxidized in the former (Fig. 1b). The consumption dynamics of atmospheric methane were indeed very similar between the 1-TF and 10-TF soils after renewing the headspace gas three times (Supplementary Fig. 1), suggesting that HAMO activity in both soils might be driven by the similar functional guilds and/or limited by the diffusion of atmospheric methane into the soil^14^,^15^,^16^. The atmospheric methane consumption rate is estimated to be ∼4.46 kg CH_4_ ha^–1^ per year by HAMO activity in the tested paddy soil. This is comparable to the activity of 4.50±0.32 kg CH_4_ ha^–1^ per year estimated for upland forest soils based on a meta-analysis of 465 methane flux data^17^, for which USCα and/or USCγ are usually found to be predominant^5^,^6^,^18^.

Nevertheless, the HAMO activity of the 1-TF soil showed a decreasing trend over time and was lost after 2 weeks (Fig. 1c). Intriguingly, this HAMO activity could be induced again on incubation with elevated methane (10,000 p.p.m.v.), despite subsequently losing HAMO activity as before (Fig. 1c). This reactivation of activity strongly indicates that the resilience and persistence of HAMO activity depends on the periodic supply and subsequent consumption of high concentrations of methane. A similar scenario of emergence and resilience was observed for the 10-TF soils. The HAMO activity decreased as rapidly as that of the 1-TF soils under ambient air, but after 10 days, weak HAMO activity was still detected, and it persisted for >2 months (Fig. 1d). It is noteworthy that most cultivated methanotrophs can oxidize atmospheric methane only after the consumption of high concentrations of methane^19^,^20^. This ability is generally lost within a few days when exposed to atmospheric methane at 1.84 p.p.m.v. in a similar manner to the exposed paddy soil in this study. This therefore suggests an important role for the known methanotrophs in the atmospheric methane uptake of periodically draining ecosystems, in which the flush-feeding of high methane typically occurs along with fluctuations in the soil water table.

### Active methanotrophs associated with soil HAMO activity

**Figure 2:**
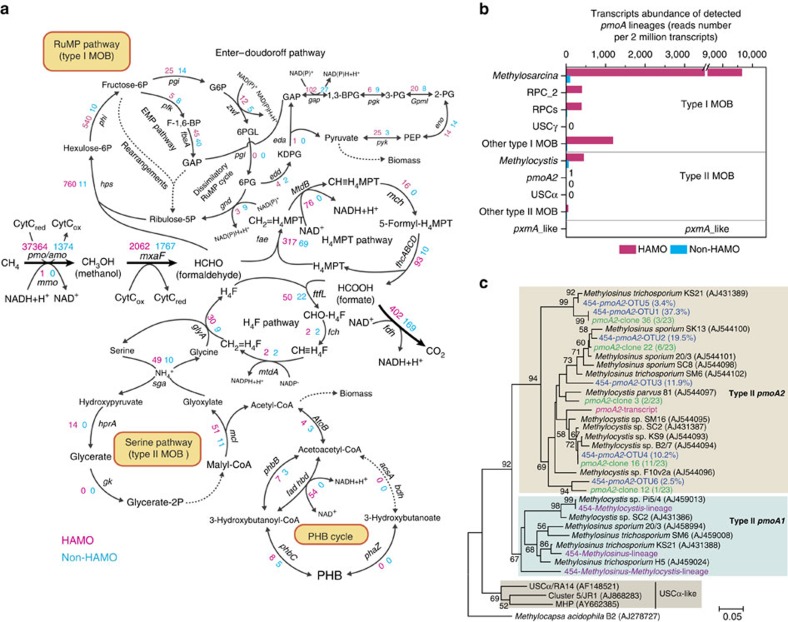
Transcript profiles of methanotrophs in paddy soil with and without high-affinity methane oxidation activity. (**a**) Methane oxidation and carbon assimilation pathways of methanotrophs that were detected in high-affinity methane oxidation (HAMO) and non-HAMO paddy soils. Gene transcript abundance was normalized as the reads number per 2 million annotated transcripts. The numbers in red and blue indicate the abundance of transcripts detected in HAMO and non-HAMO soil transcriptomes, respectively. The enzymes encoded by these genes are as follows: *acsA*, acetoacetyl-CoA synthetase; *atoB*, acetyl-CoA acetyltransferase; *bdh*, 3-hydroxybutyrate dehydrogenase; *eda*, 2-dehydro-3-deoxyphosphogluconate aldolase; *edd*, phosphogluconate dehydratase; *eno*, enolase; *fad*, 3-hydroxyacyl-CoA dehydrogenase; *fae*, formaldehyde-activating enzyme; *fbaA*, fructose-bisphosphate aldolase; *fch*, methenyltetrahydrofolate cyclohydrolase; *fdh*, formate dehydrogenase; *fhcABCD*, formylmethanofuran dehydrogenase; *ftfL*, formate-tetrahydrofolate ligase; *gap*, glyceraldehyde-3-phosphate dehydrogenase; *gk*, D-glycerate 2-kinase; *glyA*, serine hydroxymethyltransferase; *gnd*, 6-phosphogluconate dehydrogenase; *gpmI*, 2,3-bisphosphoglycerate-independent phosphoglycerate mutase; *hbd*, 3-hydroxybutyryl-CoA dehydrogenase; *hprA*, hydroxypyruvate reductase; *hps*, 3-hexulose-6-phosphate synthase; *mch*, methenyltetrahydromethanopterin cyclohydrolase; *mcl*, Malyl-CoA lyase; *mmo*, methane monooxygenase (soluble); *mtdA*, methylenetetrahydrofolate dehydrogenase; *mtdB*, methylene-tetrahydromethanopterin dehydrogenase; *mxaF*, methanol dehydrogenase; *pfk*, 6-phosphofructokinase; *pgi*, glucose-6-phosphate isomerase; *pgl*, 6-phosphogluconolactonase; *pgk*, phosphoglycerate kinase; *phaZ*, poly(3-hydroxybutyrate) depolymerase; *phbB*, acetoacetyl-CoA reductase; *phbC*, poly-beta-hydroxybutyrate polymerase; *phi*, 6-phospho-3-hexuloisomerase; *pmo*, methane monooxygenase (particulate); *pyk*, pyruvate kinase; *sga*, serine-glyoxylate transaminase; *zwf*, glucose-6-phosphate-dehydrogenase. Dotted arrows represent multiple enzymatic reactions. 1,3-BPG, 1,3-bisphosphoglycerate; EMP pathway, Embden–Meyerhof–Parnas pathway; F-1,6-BP, fructose 1,6-bisphosphate; GAP, glyceraldehyde-3-phosphate; G6P, glucose-6-phosphate; KDPG, 2-keto-3-deoxy-6-phosphogluconate; MOB, methane-oxidizing bacteria; PEP, phosphoenolpyruvate; 2-PG, 2-phosphoglycerate; 3-PG, 3-phosphoglycerate; 6PG, 6-phosphogluconate; 6PGL, 6-phospho-glucono-1,5-lactone; PHB, poly-beta-hydroxybutyrate; H_4_F, tetrahydrofolate; H_4_MPT, tetrahydromethanopterin. (**b**) Transcript abundance of the *pmoA* lineages in the HAMO and non-HAMO metatranscriptomes. Transcript abundance of each *pmoA* lineage was normalized as the read number per 2 million annotated transcripts. (**c**) The phylogeny of the detected *pmoA2* transcript (red). The tree was constructed using MEGA 4.0 based on 125 derived amino-acid sequences. Representatives of the *pmoA2* OTUs were obtained from *pmoA* gene amplicon pyrosequencing (blue) and *pmoA2* gene clone library sequencing (green), and representatives of the three pyrosequenced *pmoA1* lineages (violet) are also shown. Bootstrap values >50% (1,000 replicates) are shown at the nodes. The scale bar represents 5% sequence divergence.

We devised a MTWAS strategy by reconstructing the whole metatranscriptomes of microbial communities for comparative analysis of targeted microorganisms in the HAMO and non-HAMO soils. The expressed genes and methanotrophs responsible for the HAMO activity were characterized in 10-TF soils that displayed strong uptake of atmospheric methane for 6 days and non-HAMO soils that showed no consumption of 100 p.p.m.v. methane within an 18-day incubation period (Fig. 1). The 26.7 million high-quality messenger RNA transcript reads were taxonomically and functionally classified (Supplementary Table 1; Supplementary Data 1), using the MG-RAST (Metagenomic Rapid Annotation using Subsystem Technology) server^21^. Subsequently, the core metabolic pathways of methane oxidization and carbon assimilation were reconstructed on the basis of the detected transcripts of methanotrophic genes in the paddy soil (Fig. 2a). Gene transcripts that encode the enzymes responsible for two typical formaldehyde assimilation pathways, the ribulose monophosphate (RuMP) pathway for type I methanotrophs and the serine pathway for type II methanotrophs, were present in the HAMO soil. Both the Embden–Meyerhof–Parnas (that is, glycolysis) pathway and the collateral Enthner–Doudoroff pathway for formaldehyde assimilation were detected in the type I methanotrophs. The dissimilatory RuMP cycle for formaldehyde oxidation and RuMP regeneration was also identified, although only a few transcripts of the *gnd* gene, encoding 6-phosphogluconate dehydrogenase, were found. We also observed the expression of genes associated with the tetrahydrofolate (H_4_F) pathway, which transfers C1 units to the serine cycle for carbon assimilation in type II methanotrophs. Transcripts of the genes involved in all enzymatic reaction steps of the oxidation of methane to carbon dioxide via methanol, formaldehyde and formate were also detected. The high expression of gene transcripts, such as those associated with methanol dehydrogenase (encoded by the gene *mxaF*) and formate dehydrogenase (encoded by the *fdh* gene cluster), indicated that reducing equivalents can be continuously regenerated from the oxidization of methanol and formate and supplied to methane monooxygenase (MMO). MMO is likely to be the key enzyme that drives atmospheric methane oxidation. Expectedly, formaldehyde was oxidized to formate through a tetrahydromethanopterin-dependent pathway in the paddy soil, which was consistent with previous batch culture studies of most methylotrophs^22^. Therefore, these findings suggest that known methanotrophs likely play important roles under field conditions.

The gene transcripts affiliated with the metabolism of poly-β-hydroxybutyrate (PHB), a common carbon storage polymer in methanotrophic cells, were also identified (Fig. 2a). PHB represents an endogenous source of reducing power to methanotrophs^23^ when cells are subjected to environmental stress, such as starvation, nutrient deprivation or a lack of reducing equivalents (for example, NADH, reduced nicotinamide adenine dinucleotide)^24^. PHB accumulation often occurs under conditions of carbon excess in combination with nitrogen limitation^23^,^25^. Carbon excess in the 10-TF soils was unambiguously demonstrated by the steady decrease in methane consumption observed during the late phase of the 10-day flush-feeding (Fig. 1b). Nitrogen limitation was also evidenced by the higher expression of nitrogen-fixing genes (nitrogenase, encoded by the gene cluster *nifDKH*) and genes that transport extracellular ammonia, nitrite and nitrate into methanotrophic cells (Supplementary Fig. 2). In fact, our previous findings have shown that when the nitrogen constraint was relieved, both the methanotrophic populations and methane oxidation rates were significantly stimulated on urea amendment in the soil tested in this study^26^. Collectively, the induced stress during high-methane consumption might have a profound impact on the biomass buildup of methanotroph cells, leading to the accumulation of carbon-rich storage compounds in HAMO soils (Fig. 2a). Meanwhile, it should be noted that the expression of PHB cycle-related enzymes may be indicative of active carbon assimilation. The direct link between environmental stress and PHB accumulation remains to be experimentally established.

Among all the methanotrophic genes and gene clusters, *pmoCAB*, which encodes the particulate methane monooxygenase (pMMO), was the most enriched gene cluster with respect to the normalized abundance in the HAMO versus non-HAMO soils (Fig. 2a; Supplementary Data 1). Taxonomic classification under stringent conditions of all the putative *pmoA* transcripts (Supplementary Table 2) revealed that the most active methanotrophic lineages were *Methylosarcina* (79.7%), rice paddy cluster 2 (3.2%) (ref. 27) and rice paddy clusters (3.2%) (ref. 28) of type I methanotrophs and *Methylocystis* (3.6%) of type II methanotrophs in the HAMO soil (Fig. 2b; Supplementary Table 2). None of the 12287 *pmoA* transcripts were affiliated with the proposed atmospheric methane-oxidizing lineages USCα and USCγ (Supplementary Table 2). The absence of the USCα and USCγ lineages was substantiated through the pyrosequencing of *pmoA* amplicons from background soil, the non-HAMO soil, 1-TF and 10-TF HAMO soils (Supplementary Fig. 3; Supplementary Table 3). The deep sequencing of methanotrophic biomarkers also revealed no evidence of USCα- and USCγ-like *pmoA* genes in paddy soils across the continent, including China, Indonesia, Italy and Vietnam^29^. It thus appears unlikely that these proposed novel lineages contributed to the emergence of HAMO activity in paddy soils (Fig. 1).

A total of 441 *pmoA* transcripts could be assigned to *Methylocystis* of type II methanotrophs (Fig. 2b; Supplementary Table 2). This group has long been postulated to be putative atmospheric methane oxidizers. A recent study showed that HAMO activity in the strain *Methylocystis* sp. SC2 is catalysed by a novel *pmoCAB2* gene cluster encoding an isozyme (pMMO2) of the conventional pMMO^20^. The *pmoA2*-like transcripts were also detected in the HAMO metatranscriptome, but not in the non-HAMO soils (Fig. 2b; Supplementary Table 2). Pyrosequencing and clone library sequencing further revealed phylogenetically divergent *pmoA2* genes in the paddy soil (Fig. 2c), suggesting a greater diversity in the pMMO2-possessing type II methanotrophs than previously appreciated^20^,^30^,^31^. Cell visualization was further conducted by catalysed reporter deposition fluorescence *in situ* hybridization (CARD-FISH), using the respective group-specific 16S rRNA probes (Supplementary Fig. 4). This analysis revealed distinct morphologies typical for type I and type II methanotrophs. The result is suggestive of their roles in the emergence and resilience of HAMO activity in the paddy soil.

### Growth of known methanotrophs and HAMO activity induction

**Figure 3:**
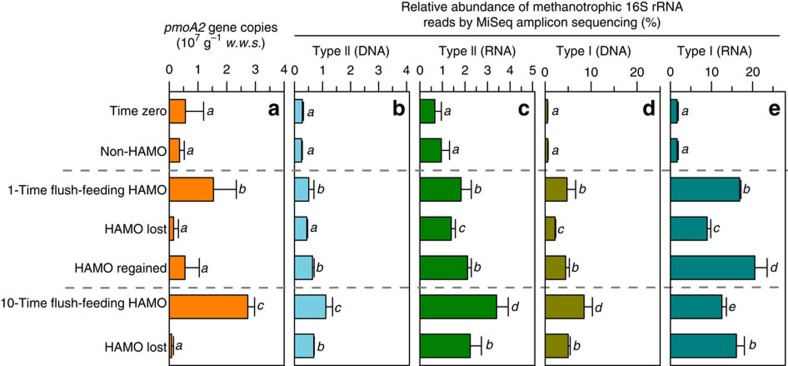
Abundances of methanotrophs associated with the emergence and resilience of high-affinity methane oxidation activity of paddy soil. (**a**) Changes in the *pmoA2* gene copy number (*pmoA2* gene-possessing methanotrophs) per gram wet weight soil (w.w.s.) as detected by qPCR. (**b**,**c**) Changes in the relative abundance of type II methanotrophs based on the Illumina MiSeq sequencing of the 16S rRNA gene (**b**) and 16S rRNA transcript (**c**) amplicons. (**d**,**e**) Changes in the relative abundance of type I methanotrophs based on the Illumina MiSeq sequencing of the 16S rRNA gene (**d**) and the 16S rRNA transcript (**e**) amplicons. Error bars represent 2 s.d. of the measurements from triplicate microcosms. Different letters above the columns indicate a significant difference (analysis of variance, *P*<0.05).

During the induction and loss of HAMO activity, the methanotrophs were quantified by real-time quantitative PCR (qPCR) of the *pmoA2* gene for pMMO2-possessing methanotrophs and by the Illumina MiSeq sequencing of the total 16S rRNA genes and their transcripts in triplicate microcosms for all other methanotrophs. There were generally higher-copy numbers of the *pmoA2* gene, as well as a higher relative abundance of the total type I and type II methanotrophs, in the HAMO soils than in the soils that lost HAMO activity (Fig. 3, Supplementary Table 4). We further found, at both the DNA and RNA levels, that type I methanotrophs were stimulated to a much greater extent than type II methanotrophs in the HAMO soils. For example, a 15.58-fold increase was observed for type I methanotrophs, but type II methanotrophs showed only a 3.95-fold increase in the 10-TF HAMO soils when compared with time zero (Fig. 3b,d). This observation was further confirmed by DNA-based stable isotope probing (DNA-SIP) of methane-C assimilating methanotrophs in 10-TF soils using ^13^CH_4_ incubation.

**Figure 4:**
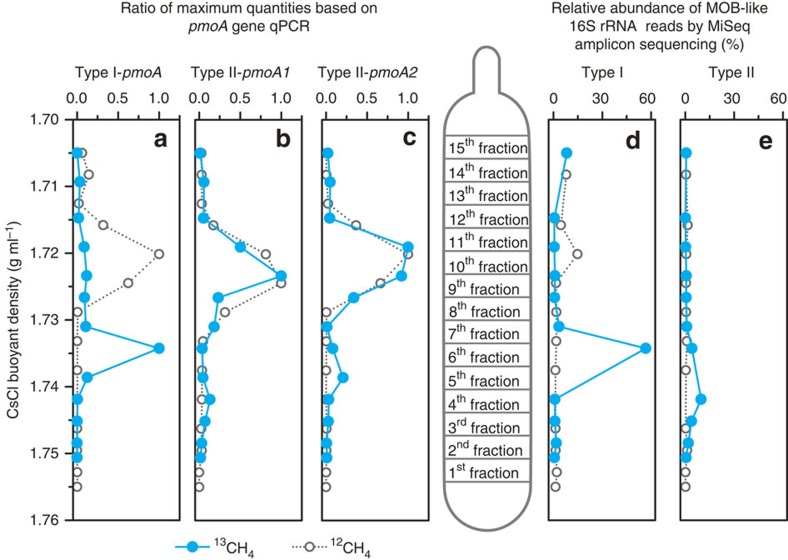
Quantitative distributions of the *pmoA* gene and methanotrophic 16S rRNA gene. Gene reads are across the entire buoyant density gradient of the DNA fractions from the HAMO soils incubated with ^13^CH_4_ and ^12^CH_4_ for 10 days. (**a**–**c**) Quantitative distribution of the *pmoA* gene from conventional type I methanotrophs (**a**), conventional type II methanotrophs (**b**) and type II methanotrophs possessing the *pmoA2* gene (**c**). The normalized data are shown as the ratio of the gene copy number in each fraction to the maximum quantities in each treatment. (**d**,**e**) Percentage distribution of type I (**d**) and type II (**e**) methanotrophs based on the Illumina MiSeq sequencing of the total bacterial 16S rRNA gene.

DNA-SIP relies on the cell division of active microorganisms grown on the ^13^C-substrates^32^. The *pmoA* gene-targeted qPCR analysis showed that the majority of type I methanotrophs were ^13^C-labelled, as their genomic DNA was detected in the heavy fractions of the fractionated DNA gradient (Fig. 4a). In contrast, only a minor fraction of the total type II methanotrophs and *pmoA2* gene-possessing methanotrophs was detected in the ^13^C-DNA fractions (Fig. 4b,c). Furthermore, based on the analysis of ∼1.3 million high-quality 16S rRNA gene reads derived from each DNA fraction (Supplementary Table 5), we also found an apparently higher proportion of type I (56.7%) than type II (9.69%) methanotrophs in the ^13^C-labelled DNA (Fig. 4d,e; Supplementary Table 5). Although it remains uncertain whether biomass carbon buildup resulted from a methane substrate or its CO_2_ production of microbial methane oxidation, these results provide strong evidence for cell division and propagation of methanotrophic communities. It has been shown that 5–15% of the biomass carbon in type I methanotrophs originates from CO_2_, and this number increases to 50% for some type II methanotrophs^33^ and 62% for others^34^. Furthermore, as shown in ig. 1b, >90% of the ^13^CH_4_ at 10,000 p.p.m.v. was rapidly consumed during the third and fourth day, and the amount of resultant ^13^CO_2_ could have been sufficiently high in support of the methanotrophic biomass buildup during the emergence of soil HAMO activity. Therefore, the faster growth observed in the ^13^C type I methanotrophs compared with the type II methanotrophs could have largely represented the difference between their life strategies as previously tested in this soil^26^, but not the substrate supply of carbon in the HAMO soils. Intriguingly, when the 1-TF HAMO soils were incubated in ambient air for ∼2 weeks until their activity was lost, the proportion of type II methanotrophs decreased only modestly (13.1%) compared with a decline of up to 55% for the type I methanotrophs (Fig. 3b,d). These results lend further support to the hypothesis that type II methanotrophs are more tolerant to environmental stress, such as substrate starvation, than type I methanotrophs^35^, suggesting that the type II methanotrophs might play important roles in the HAMO paddy soils, as previously speculated^19^,^20^,^36^.

It has been proposed that there exists a threshold amount of methane consumption for the induction of HAMO activity in soils^3^, and ∼6.67 μmol g^−1^ soil might be required in this study. A characteristic bell-shaped curve was manifest for the apparent methane oxidation rate versus the headspace methane concentration in soil microcosms (Supplementary Fig. 5). We speculate that the rapid propagation of methanotrophic cells could have occurred immediately on soil exposure to the elevated methane concentrations, leading to an increasingly higher apparent rate of soil methane oxidation (Supplementary Fig. 5). However, the optimum growth might have quickly diminished the nutrient resources when the apparent oxidation rate reached the highest value after the consumption of 6.67 μmol methane for each gram soil. Under progressively stressed conditions, the methane oxidation rate then started to decrease, while the cell growth likely ceased, resulting in the enrichment of carbon-rich compounds in methanotrophic cells for the induction of HAMO activity. It thus seems plausible that the continuous supply of methane at concentrations of 500 or 1,000 p.p.m.v. would induce HAMO activity if the cumulative consumption reached the threshold 6.67 μmol methane per g soil. Nevertheless, the threshold values may vary greatly among different types of soils^37^ and the underlying mechanism remains elusive^3^,^38^.

### Mechanism and ecological significance of soil HAMO activity

The results of this study clearly indicate the dependence of HAMO activity on the frequent supply of high concentrations of methane, providing the first experimental evidence for the hypothetical mechanism of flush-feeding in a natural setting^16^. This theoretical concept explains the atmospheric methane oxidation that occurs in periodically drained wetlands^10^. We propose that the high concentrations of methane produced within anaerobic aggregate niches during the flood season could slowly diffuse upward as a consequence of water table decline, fuelling the rapid growth of methanotrophs at the aerobic/anaerobic interface of surface soil. This low-affinity consumption further triggers the uptake of atmospheric methane for high-affinity HAMO activity by methanotrophs during drought seasons when the drained aerobic soil is exposed to the ambient air. The concurrent low and high affinity of the paddy soil assessed in this study thus provides a mechanistic basis for the oxidation of atmospheric methane in periodically draining wetlands^10^. On the basis of the HAMO activity observed in this study, the sink strength of atmospheric methane was estimated to be up to 0.4 million tons each year, considering there are >100 million hectare rice fields with periodic drainage management worldwide^39^.

HAMO activity in the paddy soil was most likely obtained under low nutrient, stress-inducing conditions. Over the course of a 10-day incubation period (Fig. 1b), the flush-feeding of 10,000 p.p.m.v. methane once a day stimulated significant methane oxidation activity at day 4. However, this low-affinity oxidation was largely slowed beginning on day 5, suggesting progressively deleterious conditions for methanotroph function. Meanwhile, the niche-deteriorating processes might also be severely aggravated owing to nitrogen constraints (Supplementary Fig. 2) and oxygen consumption in the soil. Therefore, environmental stresses resulting from rapid methanotrophic growth could be much stronger in the 10-TF than the 1-TF soils (Fig. 1a,b), leading to an accumulation of higher amounts of reductants (such as NADH) and PHB^23^,^25^. This result may explain the greater endurance of HAMO activity of >2 months in the 10-TF versus 1-TF soils (Fig. 1c,d). These endogenous reserve materials have long been presumed to act as a reducing power for the MMO enzyme in support of HAMO activity by cultivated methanotrophs. Indeed, the loss of HAMO activity is often interpreted as an exhaustion of the reducing power^19^,^36^.

The MTWAS results revealed transcriptional activity of the PHB metabolism-related genes within type II methanotrophs (Fig. 2a), suggesting that they have important roles for the induction of HAMO activity. Increasing lines of evidence have suggested that type II methanotrophs are more oligotrophic than their type I counterparts^35^, presumably conferring on the former an advantage for storage compound synthesis^23^,^40^. Interestingly, almost all *pmoA* transcripts of the type II methanotrophs were phylogenetically closely related to *Methylocystis* (Supplementary Table 2), by far the most oligotrophic methane oxidizers^19^. Some *Methylocystis* strains can even oxidize methane at atmospheric levels for up to 3 months, although growth was not observed under such low methane concentrations. Recent meta-analysis has suggested the widespread presence of *Methylocystis* in periodically draining ecosystems^13^,^41^ and in upland soils where atmospheric methane oxidation was frequently detected in the field^4^. In this study, the maintenance of HAMO activity might be partly explained by the expression of the *pmoA2* gene, which was present only in the HAMO soil (Fig. 2c). Importantly, this gene is crucial for the survival of *Methylocystis* in methane-starvation conditions^20^. Furthermore, it should be mentioned that some strains of *Methylocystis* can use acetate as a carbon source, and the reducing power derived from acetate oxidation may sustain HAMO activity^42^.

Type I methanotrophs may also play a role in atmospheric methane oxidation. A remarkably high number, up to 9,638, of *pmoA* transcripts were observed in the HAMO metatranscriptome (Fig. 2b), showing a high sequence similarity with *Methylosarcina*. Several type I methanotrophs have shown a transient ability to oxidize methane at atmospheric levels^43^,^44^. Nevertheless, type I methanotrophs are widely recognized as competitor ruderal^35^, and their *pmoA* transcripts are most likely synthesized during the rapid consumption of high concentrations of methane in HAMO soils. It also remains unknown whether *Methylosarcina* species can consume atmospheric methane^45^. Meanwhile, the genomic evidence for PHB synthesis is still missing for known type I methanotrophs^40^. However, the possibility cannot be excluded that other storage compounds may be used to support HAMO activity in the type I methanotrophs. For example, gene transcripts for glycogen metabolism were detected in the HAMO transcriptomes (Supplementary Table 6). Considering all tested type I methanotrophs store glycogen, they should also be able to supply reducing equivalents to pMMO under starvation conditions by using this storage compound. In addition, intermediate metabolites, such as methanol or formate, may also act as reducing equivalents to sustain HAMO activity, as shown in pure culture^46^,^47^. The use of a selective inhibitor to differentiate methanotrophs at high taxonomic resolution may therefore hold great promise for quantifying the relative contributions of different known methanotrophs to atmospheric methane oxidation in soils.

Taken together, our results demonstrate that the induction of atmospheric methane oxidation occurred only after the consumption of high concentrations of methane in paddy soil. However, the induced activity was lost unless the soil was periodically flushed with high concentrations of methane. On the depletion of flush-feeding methane, the methanotrophs may rely on an endogenous storage compound, such as PHB, as a co-substrate to maintain cell viability while consuming atmospheric methane. The concurrent high- and low-affinity methane oxidation in the paddy soil was catalysed by microbial guilds within the known methanotrophic lineages rather than the putative atmospheric methane oxidizers of USCα and USCγ. The emergence and resilience of HAMO activity in the paddy soil provides a mechanistic basis for a better understanding of methane turnover in periodically draining ecosystems and necessitates a re-assessment of atmospheric methane oxidation in terrestrial ecosystems to reduce the uncertainty of the global methane budget.

## Methods

### Site description of the soil samples

Soil samples were collected from a paddy field (119° 42′ 0′′ E and 32° 35′ 5′′ N) in Yangzhou City of the Jiangsu province, a typical subtropical agricultural region in China used for rice–wheat rotation^48^. Soil cores (0–15-cm depth) were collected by a steel corer with three replicates after the rice harvest, homogenized by passing through a 2-mm meshed sieve and then stored at 4 °C until further use. The soil characteristics were as follows: maximum water holding capacity, 55%; total organic carbon, 15 g kg^−1^; total *N*, 1.59 g kg^−1^; total *P*, 1.23 g kg^−1^; and pH 7.4 (ref. 26).

### Microcosm construction with the flush-feeding of methane

Before microcosm construction, the soil was preincubated at 60% water holding capacity and 28 °C in darkness for 4 days under ambient air conditions. Then, aliquots (0.5 g, wet weight) of the soil were placed into sterile 2-ml centrifuge tubes with 1 ml RNA*later* (Ambion) as the initial sample (time zero) and stored at −20 °C until nucleic acid extraction^49^. Microcosms were constructed by adding 5 g preincubated paddy soil to 120-ml serum vials capped with butyl rubber stoppers as described previously^26^,^50^. Two treatments were performed, including 1-TF and 10-TF of methane at high concentrations. The 1-TF microcosms had initial methane concentrations of 10,000, 1,000, 500, 100 and 2 p.p.m.v. (ambient air). The 10,000 p.p.m.v. CH_4_ was renewed once a day in the 10-TF microcosms for 10 days. The methane concentration in the headspace of each vial was measured by GC-FID (Shimadzu GC12-A, Japan)^51^. The HAMO activity referred to the consumption of headspace methane in microcosms below 2 p.p.m.v. The headspace gas was flushed with ambient air to monitor the persistence of the HAMO activity. When the soil could not oxidize the atmospheric methane, it was exposed again to 10,000 p.p.m.v. CH_4_, and the HAMO activity was determined three times. The HAMO activity of the 10-TF soils was also monitored using the same method as the 1-TF soils after flushing the headspace gas with ambient air. Destructive soil samplings were performed when the soil gained or lost HAMO activity. The empty vials with 10,000 p.p.m.v. CH_4_ were also monitored over the experiment period to guarantee the absence of gas leaks. All treatments were conducted at least in triplicate.

The HAMO activity of the paddy soil was assessed based on the amount of atmospheric CH_4_ that can be oxidized in the first 3 or 3.5 h (data from Supplementary Fig. 1). The CH_4_ concentration in the 120-ml vials decreased at a rate of 0.18584, p.p.m.v. h^−1^ (the average value of that in 1-TF and 10-TF treatments), and the soil covers an area of ∼1.256 × 10^−3^ m^2^ with a depth of ∼0.5 cm. It is assumed that in the rice field, the HAMO activity of the 2 cm surface soil could be induced and that the methane density is 0.7163, g l^−1^ at 25 °C with a pressure of 1 atm. The estimated atmospheric methane oxidation rate of the HAMO soils could be up to 305.22 μg per day per m^2^, which is equivalent to 4.46 kg ha^−1^ per year.

### Nucleic acid isolation

The total nucleic acid content was extracted from soil samples as previously described^52^ with a slight modification. In brief, soil samples stored at −20 °C in RNA*later* were thawed on ice, resuspended and pelleted at 20,000 *g* for 1 min. Then, the pellets were mixed with 0.5 g glass beads (0.5 mm: 0.1 mm=3:2, Sigma) and processed for cell lysis using two rounds of bead-beating using acidic lysis buffers. The resulting supernatant was successively extracted using water-saturated phenol (pH 4.5), phenol–chloroform–isoamyl alcohol (25:24:1, (vol/vol/vol), pH 4.5) and chloroform–isoamyl alcohol (24:1, (vol/vol), pH 5.5) extraction. Nucleic acids were precipitated using two volumes of PEG–NaCl (30% PEG-6000, 1.6 M NaCl), washed with 400 μl 70% ethanol and resuspended in 50 μl nuclease-free H_2_O, of which 10 μl was 10-fold diluted and used for DNA-based amplification. For the RNA isolation, the rest of the extract was treated with recombinant DNase I (Takara) to digest the DNA and then purified using the RNeasy Mini Kit (Qiagen, Germany). DNA contamination was ruled out by PCR amplification using the universal 16S rRNA gene primers (515F, 5′-GTGCCAGCMGCCGCGG-3′; 907R, 5′-CCGTCAATTCMTTTRAGTTT-3′) (ref. 53) with purified RNA as a template. The total RNA was converted to complementary DNA (cDNA) using a PrimeScript 1st Strand cDNA Synthesis Kit (TaKaRa) and random hexamers, and was stored at −20 °C for amplicon sequencing.

### Metatranscriptomic analysis of the paddy soil

Metatranscriptomes were obtained from the HAMO (10-TF soils that displayed strong uptake of atmospheric methane for 6 days) and non-HAMO (100 p.p.m.v. methane-amended soil that showed no methane oxidation within an 18-day incubation period) soils (marked in Fig. 1). The total RNA of each soil sample from triplicate microcosms was extracted as described above and pooled together. Then, the rRNA was depleted using the Ribo-Zero Magnetic Kit (Bacteria; Epicentre). The cDNA was obtained using the Superscript Double-Stranded cDNA Synthesis kit (Invitrogen) and a random hexamer primer, and then fragmented to ∼200 bp using a Covaris M220. The library was constructed using the TruSeq DNA Sample Prep Kit, and the sequencing was performed on an Illumina HiSeq2000 platform.

The raw FASTQ data files were submitted to MG-RAST 3.0 for taxonomic and functional annotation^21^. The taxonomic affiliation of the messenger RNA transcripts was determined using the ‘Best Hit Classification' method against the M5NR protein database with the following parameters: average e-value less than or equal to 1e−5, average align length ≥30 aa and average % identity ≥60. Transcripts affiliated to the methanotrophs were selected to workbench, and then the Subsystem database was used to make a functional annotation of these transcripts (with an average e-value less than or equal to 1e−10, average align length ≥30 aa and average % identity ≥60). The transcript abundance of each function category in the HAMO and non-HAMO metatranscriptomes was normalized as the reads number per million total transcripts annotated by the Subsystem database before the reconstruction of the metabolic pathways of methane and nitrogen.

All of the putative *pmoA* gene transcripts in the HAMO and non-HAMO metatranscriptomes were selected to infer the taxonomic identities of the active methanotrophs. This process was conducted as follows. (1) The preparation of a database containing 6,628 *pmoA* and *pmoA*-related sequences of both cultured methanotrophic strains and as-yet-uncultured methanotrophic ecotypes, such as USCα and USCγ, was completed as previously described^54^. (2) The screening of *pmoA* gene transcripts in the soil metatranscriptomes was accomplished. The gene function annotation of the two metatranscriptomes in MG-RAST was first performed using the GenBank nr database as the annotation source (e-value less than or equal to 1e−5, average align length ≥30 aa and average % identity ≥60); and then, all the putative *pmoA* sequences (containing ‘methane monooxygenase' in their functional annotation) were selected and downloaded in fasta format. (3) A BLAST search was completed for the putative *pmoA* sequences against the *pmoA* database (step 1) and then classified them to known *pmoA* lineages using the lowest common ancestor method in MEGAN^55^ as described previously^54^. The transcript abundance of each *pmoA* lineage in the HAMO and non-HAMO metatranscriptomes was normalized as the reads number per 2 million transcripts that were annotated using the GenBank nr database.

### Stable isotope probing of active methanotrophs in paddy soil

To monitor the active methanotrophs during the emergence and resilience of HAMO activity, DNA-SIP incubation with 10,000 p.p.m.v. ^13^CH_4_ and ^12^CH_4_ was performed as the labelled and control treatment, respectively. The destructive sampling was performed at the 10-TF microcosms. Genome DNA was extracted using the FastDNA spin kit for soil (MP Biomedicals, Cleveland, OH, USA) according to the manufacturer's instructions. DNA-SIP fractionation was performed as previously described^26^. Up to 14 gradient fractions were generated, and a refractive index measurement of each fraction was determined using an AR200 digital hand-held refractometer (Reichert, Inc., Buffalo, NY, USA). The fractionated DNA was recovered by PEG-6000 precipitation and resuspended in 30 μl TE buffer, and then used for MiSeq sequencing and real-time qPCR.

### Pyrosequencing of *pmoA* gene

Multiplex amplicon pyrosequencing of the *pmoA* gene was used to generate a comprehensive assessment of the methanotrophic community in the studied paddy soil. DNA samples from the time zero, non-HAMO, 1-TF HAMO and 10-TF HAMO soil were selected for PCR amplification using the barcode primer pair A189f/A682r (ref. 56). Pooled amplicons from the triplicate microcosms were mixed at an equimolar ratio and sequenced on a Roche 454 GS FLX Titanium sequencer (Roche). A raw sequence file was processed with the commands ‘trim.seqs' (minlength=400 and qaverage=30) and ‘split groups' using the Mothur software (version 1.33.3) for quality control and sample splitting^57^. These reads were then processed using the online version of FunGene Pipeline^58^ to check the chimera using USEARCH 6.0 (ref. 59) and to correct frameshifts using FramBot^60^. High-quality *pmoA* sequences of each sample were then classified as known *pmoA* groups or lineages as described above. *pmoA* sequences of a specific group or lineage were selected and downloaded from MEGAN and were clustered into a species-level operational taxonomic unit (OTU) based on their deduced amino-acid sequences by FunGene Pipeline with a distance cutoff of 0.07 (ref. 61). One representative of each lineage (a representative of the dominant OTU of each lineage) and several representatives of dominant OTUs of the *pmoA2* gene were extracted and used for phylogenetic analysis. A *pmoA* phylogenetic tree was constructed using the neighbour-joining method in MEGA 4.0 (ref. 62) with bootstrapping of 1,000 replicates.

### Clone library construction of the *pmoA2* gene

A clone library of the *pmoA2* gene was also constructed in the 10-TF soils. The pmoA206f/pmoA703b primer pair was used to amplify the *pmoA2* genes as previously described^30^. Triplicate amplicons were pooled, ligated into the pGEM-T vector (Promega, Fitchburg, WI, USA) and transformed into competent DH5α cells. Twenty-three *pmoA2* sequences were obtained by Sanger sequencing of the randomly selected positive clones. OTU clustering and representative picking were also performed through the FunGene Pipeline.

### Quantification of the *pmoA* gene

The abundance of the conventional *pmoA* gene of type I and type II methanotrophs in the fractioned DNA was quantified by qPCR using primer pairs A189F/Mb601R and II223F/II646R, respectively, as previously described^63^. The copy numbers of the *pmoA2* gene in the different methane-treated soils and in the fractioned DNA were also determined using the primer pair pmoA206f/pmoA703b. The reactions were performed in a 20-μl mixture containing 10.0 μl SYBR Premix Ex Taq (Takara), 0.5 μM each primer and 1 μl of DNA template. All qPCR assays were performed in triplicate on a CFX96 Optical Real-Time Detection System (Bio-Rad, Laboratories Inc., Hercules, CA, USA). The respective standards were obtained by a 10-fold dilution series of plasmids containing the respective *pmoA* gene fragments. The amplification efficiencies for all the three primer pairs were between 81.5 and 94.1%, with *R*^2^ values of 0.993–0.998.

### MiSeq sequencing of the 16S rRNA genes and their transcripts

The microbial community structure in the different sampling points was assessed by Illumina MiSeq sequencing of the 16S rRNA genes and transcripts from the triplicate microcosms. The microbial community composition across the DNA-SIP fractions was also assessed. The 515F/907R primer pair was used for the amplification of the V4–V5 region of the 16S rRNA gene, and a 12-bp sample-specific adaptor sequence was attached to the 5′ end of 515F. PCR was conducted using *TaKaRa Taq* Hot Start Version (TaKaRa, Japan) in a total volume of 50 μl with 1.5 U *TaKaRa Taq* HS, 4 μl dNTP mixture (10 mM), 5 μl 10 × PCR buffer, 0.5 μl bovine serum albumin (20 mg ml^−1^, Takara), 1 μl of each primer (10 μM) and 1 μl template DNA. The PCR conditions were as follows: 2 min at 94 °C; 30 cycles consisting of 30 s at 94 °C, 30 s at 60 °C and 45 s at 72 °C; with a final extension step at 72 °C for 5 min All amplicons were verified on 1.2% (W/V) agarose gels stained with Goldview, purified using a MiniBEST DNA Fragment Purification Kit Ver.3.0 (TaKaRa), quantified by a NanoDrop ND-1000 spectrophotometer and mixed at an equimolar ratio. The library was constructed using TruSeq Nano DNA LT Sample Prep Kit Set A (24 samples), and sequencing was performed using a MiSeq Reagent Kit v3 (600 cycles) following the manufacturer's protocol.

Mothur software (version 1.33.3) was used for read merging and quality filtering. Pair-end reads were merged using the ‘make.contigs' command (deltaq=5), and the ‘trim.seqs' command was used for tags and primer removal and length trimming (leaving sequences between 370 and 380 bp in length). The ‘classify.seqs' command was used for the taxonomic classification of the high-quality sequences (total 3,924,056) with a cutoff of 60% using the parameter settings of ‘Wang' method^64^.

### CARD-FISH

CARD-FISH was performed to identify the type I and type II methanotrophs, and total bacteria as previously described^65^. Cells were extracted in triplicate from 1.0 g paddy soils as previously described^66^ with some modifications. In brief, the soil was first suspended in 10 ml 0.2% sodium pyro-phosphate and homogenized by vortexing for 15 min, and then the soil homogenate was layered onto the same volume of saturated sucrose solution (1.33 g ml^−1^). After being centrifuged at 5,500*g* for 2 min, the upper sucrose fraction containing the bacterial cells was poured into a new 15-ml polyallomer centrifuge tube, diluted with 1/3 volume of 0.8% NaCl and centrifuged at 12,000*g* for 10 min at 4 °C. The cell pellet was then resuspended in 4 ml 0.8% NaCl and transferred to a 10-ml sterilized tube containing 4 ml Nycodenz solution (1.310 g ml^−1^). After being centrifuged at 9,500*g* for 25 min at 4 °C, the ‘cell layer' between Nycodenz and the aqueous layer was recovered, washed and then pelleted by centrifugation at 12,000*g* for 10 min at 4 °C. The cell pellet was resuspended in 0.2 ml PBS and used for CARD-FISH analyses and 4,6-diamidino-2-phenylindole staining. In brief, after being fixed with formaldehyde (2% final concentration) at 25 °C for 45 min, the cell suspensions were filtered onto 0.22 μm polycarbonate membranes (Millipore). Then, the filters were washed with 1 × PBS, air-dried, treated with lysozyme (10 mg ml^−1^) for 30 min at 37 °C and washed again with water. The filters were then hybridized with horseradish peroxidase-labelled probes for 3 h at 46 °C in the hybridization solution. Probes EUB I-III^67^, Mγ669 and Mα450 (ref. 68) were used for the 16S rRNA-targeted hybridization of the total bacteria and type I and type II methanotrophs, respectively. Probe NON338 was used as a negative control^69^. The filters were then washed with washing buffer (30 min at 48 °C), transferred into an amplification buffer containing 0.0015% H_2_O_2_ and 1 μg ml^−1^ Oregon Green-labelled tyramides for 30 min at 46 °C, washed with 1 × PBS and stained with 4,6-diamidino-2-phenylindole (50 ng ml^−1^) for 10 min at 25 °C in the dark. After embedding in a mixture of Citifluor/Vectashield, the filters were viewed and photographed under an epifluorescent microscope (Nikon Eclipse 80i).

### Data availability

All sequence data that support the findings of this study have been deposited in the following public repositories. The raw amplicon sequence data sets for both the 16S rRNA and *pmoA* genes have been deposited in the European Nucleotide Archive (ENA) under the accession code ERP008876. Metatranscriptomic data have been deposited in MG-RAST with accession codes 4553284.3 and 4553285.3. All *pmoA2* gene sequences obtained from clone library sequencing have been deposited in GenBank with accession code KP218949 to KP218971. Source data for Figures (Figs 2, 3, 4) are provided with the article in the Supplementary Information files. The authors declare that all the relevant data that supporting the findings of this study are available within the article and its Supplementary Information on request.

## Additional information

**How to cite this article:** Cai, Y. *et al*. Conventional methanotrophs are responsible for atmospheric methane oxidation in paddy soils. *Nat. Commun.* 7:11728 doi: 10.1038/ncomms11728 (2016).

## Supplementary Material

Supplementary Information Supplementary Figures 1-5 and Supplementary Tables 1-6.

Supplementary Data 1 Transcripts affiliated to methanotrophs in HAMO and Non-HAMO metatranscriptomes. Subsystem in MG-RAST was used for gene function annotation (with average e-value no more than -10, average align length no less than 30 aa, and average % identity no less than 60). Transcripts abundance was normalized to present as reads number per million function-known transcripts. Fold changes [(% in HiMO)/(% in Non-HiMO)] of these gene transcripts in HAMO and non-HAMO metatranscriptomes are shown as well.
