# Supplementary material for: Conventional methanotrophs are responsible for atmospheric methane oxidation in paddy soils
Source: Nat Commun. 2016 Jun 1;7:11728. doi: 10.1038/ncomms11728 (PMC4895445; doi:10.1038/ncomms11728)
Supplement: Supplementary Information — Supplementary Figures 1-5 and Supplementary Tables 1-6. [file ncomms11728-s1.pdf]

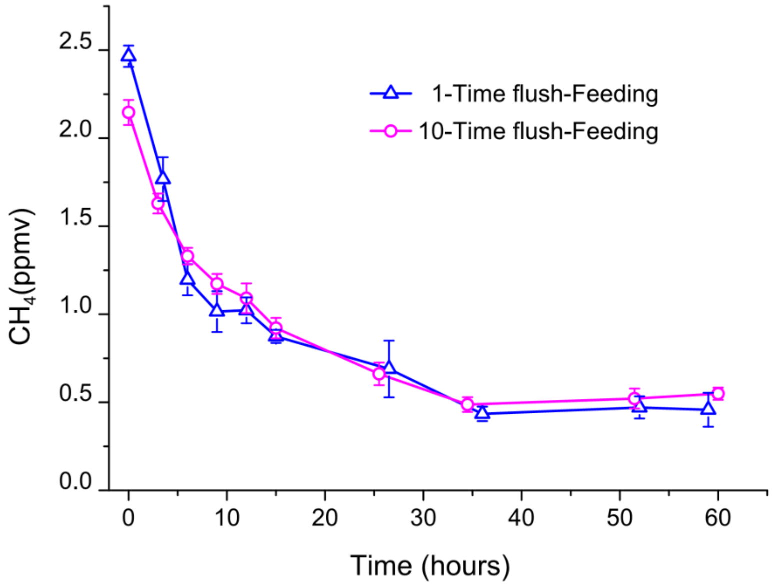

Supplementary Figure 1. **High-affinity methane oxidation (HAMO) dynamics of soils with added methane at 10000 ppmv for 1 time and 10 times.** After the complete consumption of 10000 ppmv methane, the measurement was conducted by renewing the headspace with ambient air. Error bars represent 2 s.d. of the measurements from the triplicate microcosms. No significant difference were found between these two curves ($P > 0.05$, paired samples t test).

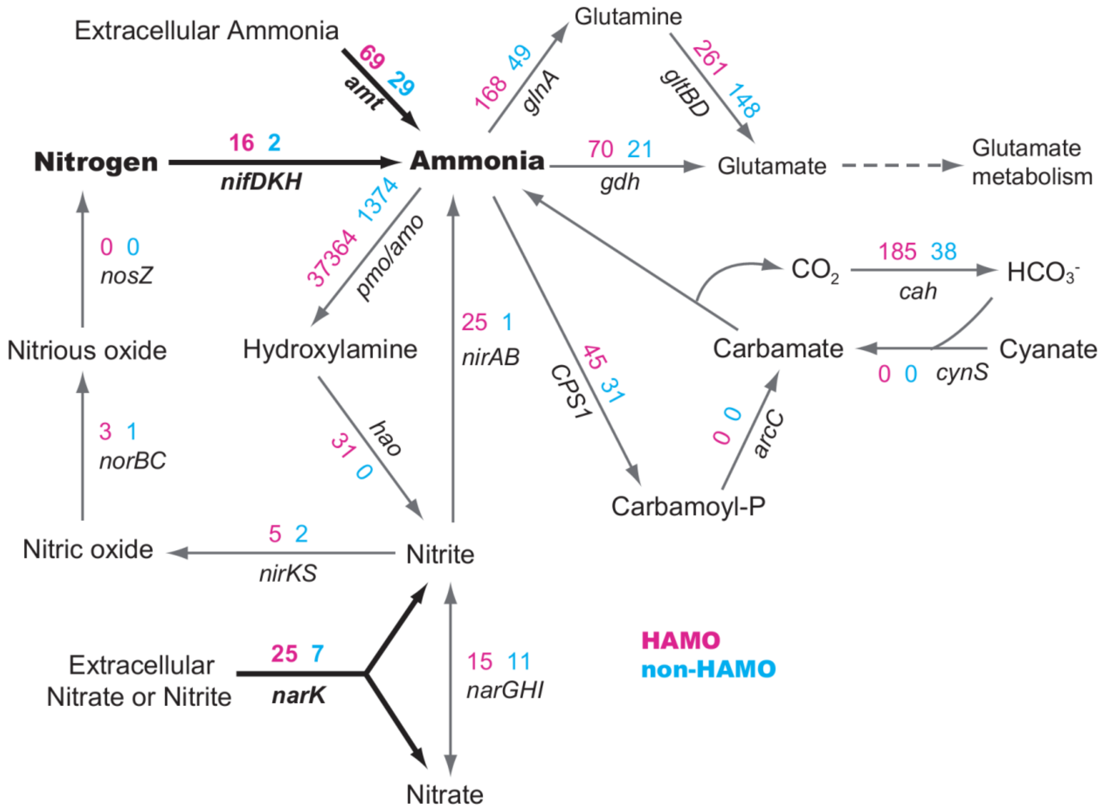

Supplementary Figure 2. **Reconstruction of the nitrogen metabolic pathway based on methanotrophic transcripts detected in paddy soils with and without HAMO activity.** The numbers in red and blue indicate the abundance of transcripts detected in the HAMO and non-HAMO transcriptomes, respectively. The gene transcript abundance was normalized as the reads number per million transcripts annotated within the Subsystem in MG-RAST. The enzymes or proteins encoded by these genes areas follows: *amt*, ammonium transporter; *arcC*, carbamate kinase; *cah*, carbonic anhydrase; *CPS1*, carbamoyl-phosphate synthase (ammonia); *cynS*, cyanate lyase; *gdh*, glutamate dehydrogenase; *glnA*, glutamine synthetase; *gltBD*, glutamate synthase (NADPH); *hao*, hydroxylamine dehydrogenase; *narGHI*, nitrate reductase; *narK*, nitrate/nitrite transporter; *nifDKH*, nitrogenase (molybdenum-iron); *nirAB*, ferredoxin---nitrite reductase and nitrite reductase (NADH); *nirKS*, nitrite reductase (NO-forming); *norBC*, nitric oxide reductase; *nosZ*, nitrous oxide reductase; *pmo*/*amo*, methane/ammonia monooxygenase. The uptake steps of extracellular nitrogen are indicated by the bold arrows.

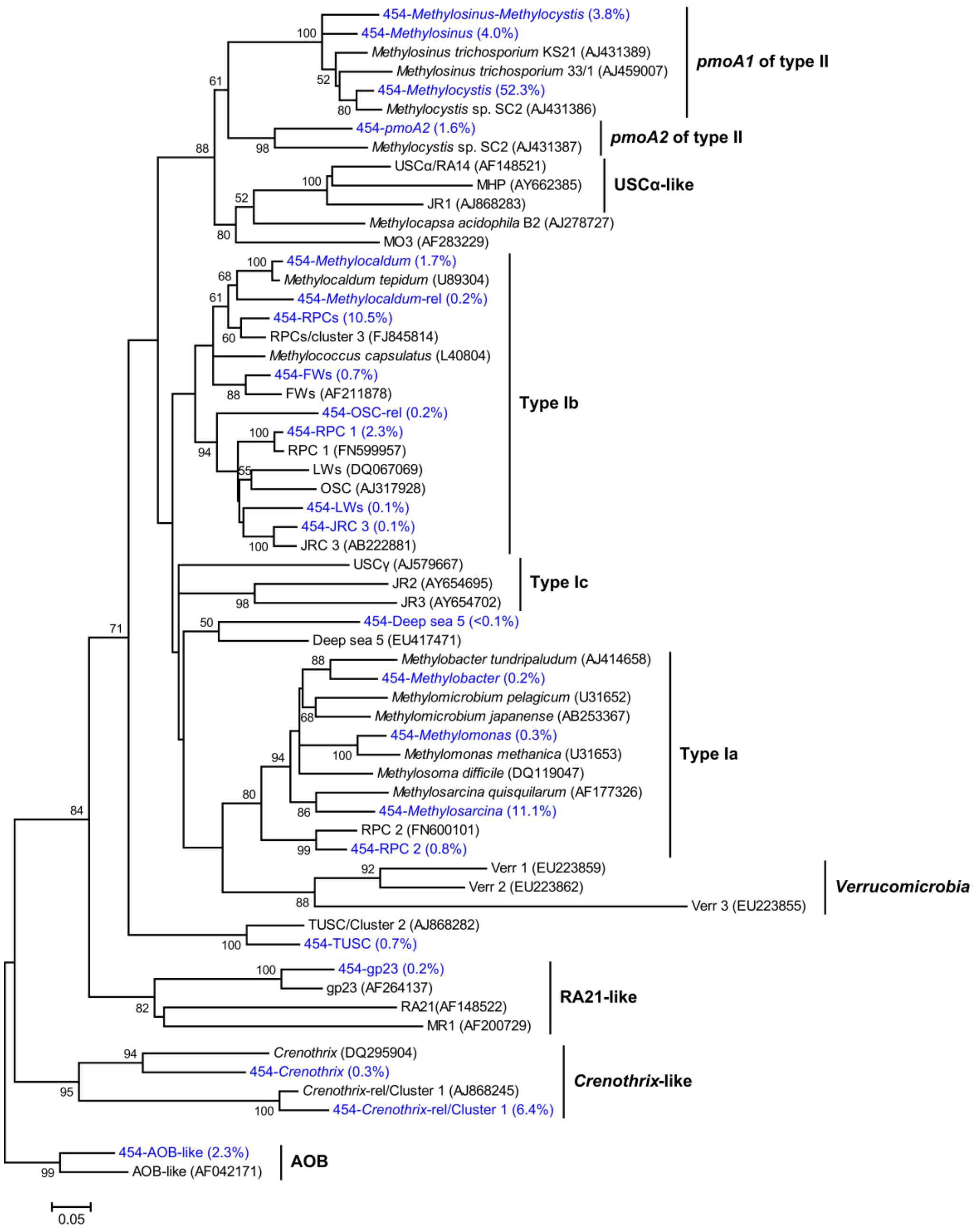

Supplementary Figure 3. Phylogeny of the *pmoA* genes detected in the paddy soil. The Neighbor-joining tree was constructed using the MEGA 4.0 software package based on 125 deduced amino acid sequences. Representatives of the lineages detected in the paddy soil samples (time zero, non-HAMO, 1-Time Flush-Feeding and 10-Time Flush-Feeding) by *pmoA* amplicon pyrosequencing are shown in blue. *PmoA-like* sequences affiliated with AOB (ammonia-oxidizing bacteria) were used as the outgroup. Only bootstrap values greater than 50% (1,000 replicates) are shown at the nodes. The scale bar represents 5% sequence divergence. The following abbreviations of lineages without cultured representatives are used: FW, freshwater sediment of Lake Wintergreen, Michigan, USA; JR, Jasper Ridge, California, USA; JRC, Japanese rice cluster; LW, sediment of Lake Washington, USA; MHP, Moor House peat, England; MR, forest near Marburg, Germany; RA, Rold Forest, Denmark; RPC, rice paddy cluster; TUSC, tropical upland soil cluster; USC, upland soil cluster.

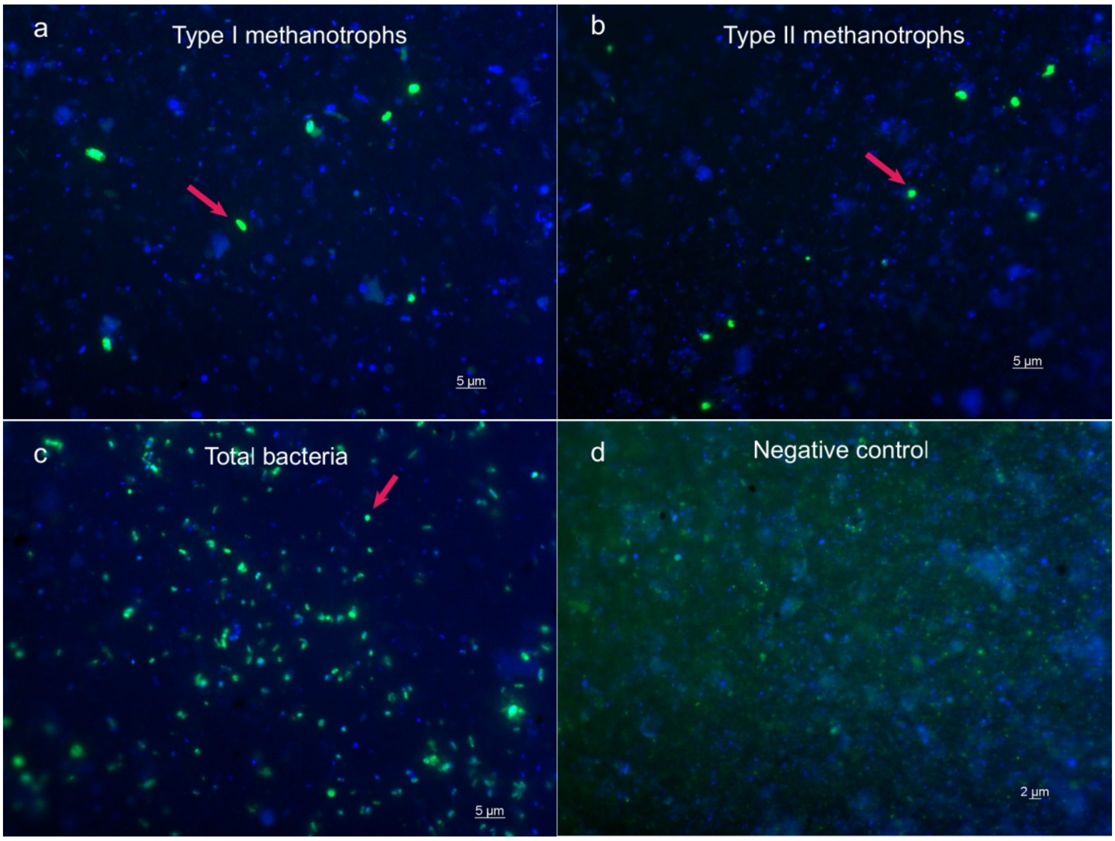

Supplementary Figure 4. **Photomicrographs of CARD-FISH (catalyzed reporter deposition-fluorescence in situ hybridization) and DAPI (4', 6-diamidino-2-phenylindole) stained cells.** Cells were derived from the 10-Time Flush-Feeding paddy soil and show high-affinity methane oxidation. CARD-FISH stained (green) type I methanotrophs (a), type II methanotrophs (b) and total bacteria (c) are shown using the group specific probes Mγ669, Mα450 and EUB I-III, respectively. Negative control of CARD-FISH using a nonspecific binding probe NON338 (d). The DAPI stained cells are shown in all four panels in blue.

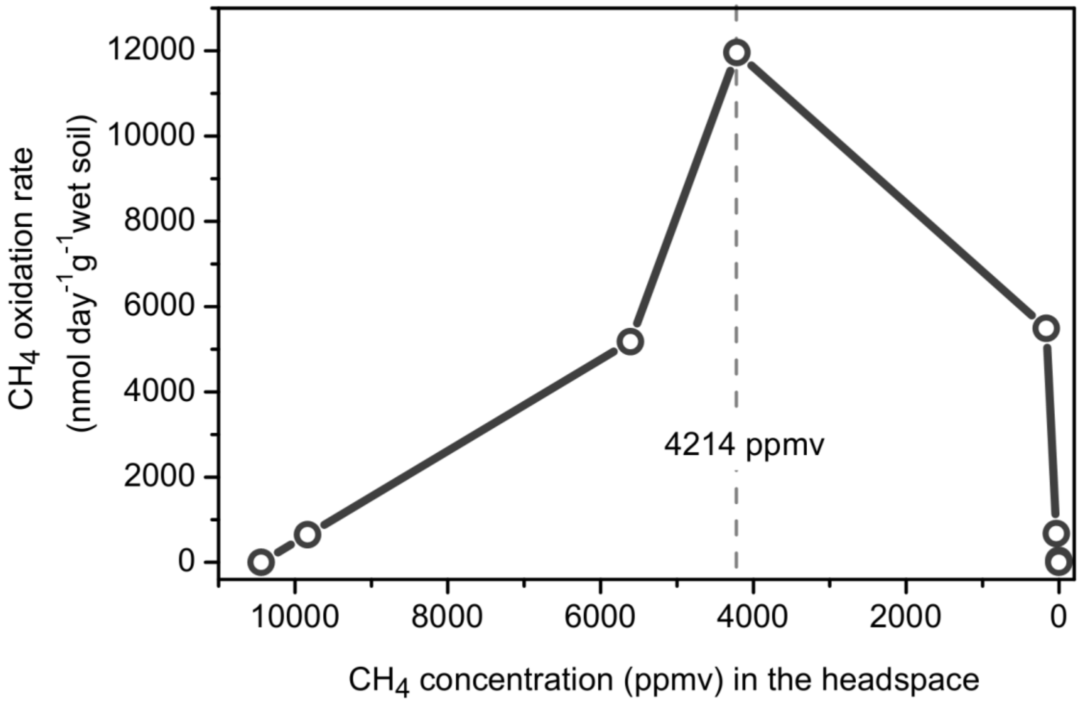

Supplementary Figure 5. **Change in the apparent methane oxidation rate versus the headspace methane concentration in soil microcosms amended with 10000 ppmv methane.** The apparent rate of soil methane oxidation was calculated using the following equation: the methane oxidation rate = the amount of methane consumed for each gram of soil divided by time between the two neighboring measurement points. The simulated highest rate of methane oxidation was indicated by the vertical dashed line.

Supplementary Table 1. Summary of HAMO (high-affinity methane oxidation) and non-HAMO metatranscriptomes

	HAMO	Non-HAMO
Raw sequences (paired)	28,553,977	36,444,367
Sequence length of raw sequences (bp)	101	101
Submitted sequences (pair merged)	22,480,376	30,507,648
Artificial duplicate sequences	10,487,831	15,797,270
Sequences passed quality control	11,992,545	14,710,378
Mean sequence length after quality control (bp)	161 ± 19	159 ± 18
Predicted protein features	9,638,854	11,697,063
Sequences annotated by M5NR database[a]	4,854,771	5,337,521
Sequences annotated by GenBank nr database[b]	2,009,255	2,222,255
Sequences assigned to Subsystems[c]	1,059,586	1,050,766

a, Cutoff setting of taxonomic classification using the 'Best Hit Classification' tool: Max. e-Value: 1e-5, Min. % Identity: 60, Min. Alignment Length: 30.

b, Cutoff setting of function gene annotation: Max. e-Value: 1e-5, Min. % Identity: 60, Min. Alignment Length: 30.

c, Cutoff setting of function gene category annotation using the ‘Hierarchical Classification’ tool: Max. e-Value: 1e-10, Min. % Identity: 60, Min. Alignment Length: 30.

Supplementary Table 2. Taxonomic classification of *pmoA* transcripts

pmoA group or lineage based on BLAST and MEGAN[a]			Reads abundance	
			HAMO	Non–HAMO
Proposed atmospheric methane oxidizing groups				
Type II	Type IIb	*pmoA*2	1[b]	–
		USCα	–	–
Type I	Type Ib	USCγ	–	–
Non Atmospheric methane oxidizing groups				
Type I	Type Ia	*Methylobacter*	244	5
		Methylomonas	4	1
		Methylosarcina	9638	93
		Methylomicrobium japanense	341	–
		RPC–2	392	2
		LP20	3	–
		Unclassified type Ia	169	–
	Type Ib	FWs	107	4
		LWs	1	1
		Methylocaldum	32	5
		Methylocaldum–rel	4	–
		Methylothermus	125	3
		RPC–1	52	–
		RPCs	383	16
		Lake_cluster_2	27	–
		Deep sea 5	13	–
		Unclassified type Ib	58	1
	Type Ic	JR3	–	1
		JR3-rel	1	–
Type II	Type IIa	*Methylocystis*	441	58
		Methylosinus	4	1
		Methylosinus/Methylocystis	2	1
		Unclassified type IIa	42	6
pxmA–like	Crenothrix–like	Crenothrix	1	1
	M84-P105	M84-P105	3	–
Total			12088	199

a, LCA Parameters used in MEGAN is Min Support: 1, Min Score: 150, Top Percent: 1.0, Win Score: 50.0.

b, Reads abundance was normalized as the reads number for each 2 million transcripts annotated by GenBank nr database.

–, not determined.

Supplementary Table 3. Taxonomic classification of the *pmoA* gene sequences obtained by pyrosequencing

pmoA group or lineage based on BLAST and MEGAN[a]			High-quality reads abundance			
			Time zero	Non–HAMO	1-TF[b] HAMO	10-TF[c] HAMO
Proposed atmospheric methane oxidizers						
Type II	Type IIb	*pmoA*2	41	39	20	19
		USCα	–	–	–	–
Type I	Type Ib	USCγ	–	–	–	–
Non-atmospheric methane oxidizers						
Type I	Type Ia	*Methylobacter*	5	1	2	4
		Methylomonas	6	6	11	3
		Methylosarcina	7	8	284	543
		RPC–2	–	–	22	37
		Unclassified type Ia	–	–	–	1
	Type Ib	FWs	10	7	15	13
		JRC–3	1	–	4	1
		LWs	–	–	1	–
		Methylocaldum	40	36	30	22
		Methylocaldum–rel	1	2	6	9
		RPC–1	33	15	104	43
		RPCs	87	106	344	240
		Deep sea 5	1	–	–	1
		Unclassified type Ib	12	4	21	5
Type II	Type IIa	*Methylocystis*	770	490	1,184	1,500
		Methylosinus	122	78	75	29
		Methylosinus/Methylocystis	72	68	47	43
		Unclassified type IIa	35	9	34	18
	Unclassified type II		–	–	1	–
pxmA–like	Crenothrix–like	Crenothrix	13	3	2	–
		Crenothrix–rel	268	65	121	33
	RA21–like	gp23	2	3	4	1
	TUSC–like	TUSC	19	16	8	7
		TUSC-rel	1	–	–	–
AOB–like			70	54	41	12
Not-assigned			4	1	2	1
No-hits			–	–	–	1
Total			1,620	1,011	2,383	2,586

a, LCA Parameters used in MEGAN is Min Support: 1, Min Score: 400, Top Percent: 5.0, Win Score: 50.0, Min Complexity: 0.0.

b, 1-Time flush-Feeding.

c, 10-Time flush-Feeding.

–, not determined.

Supplementary Table 4. High-throughput sequencing of 16S rRNA genes and transcripts in paddy soils with and without HAMO (high-affinity methane oxidation) activity

Treatment	Replicate	Number of high quality 16S rRNA Reads		Type I methanotrophs (%)		Type II methanotrophs (%)	
		DNA	RNA	DNA	RNA	DNA	RNA
Time zero	R1	43,413	26,491	0.6	1.3	0.3	0.4
	R2	71,311	70,707	0.5	1.8	0.3	0.8
	R3	21,252	58,276	0.5	1.8	0.3	0.9
Non-HAMO	R1	26,219	109,479	0.6	1.7	0.3	1.3
	R2	56,382	4,470	0.5	1.3	0.2	1.0
	R3	14,196	25,331	0.6	1.8	0.3	0.6
1-TF[a] HAMO	R1	47,873	90,741	6.9	17.0	0.7	2.2
	R2	13,436	82,442	3.5	17.1	0.4	2.0
	R3	22,330	53,078	3.9	16.5	0.4	1.3
1-TF HAMO-lost	R1	45,843	53,416	2.1	9.5	0.4	1.3
	R2	115,066	66,645	2.3	9.5	0.4	1.2
	R3	93,542	59,258	2.0	8.0	0.5	1.6
1-TF HAMO-regained	R1	39,175	76,952	4.3	17.7	0.7	2.3
	R2	35,977	98,852	3.7	20.4	0.6	2.0
	R3	45,607	89,379	5.3	23.7	0.7	2.1
10-TF[b] HAMO	R1	64,339	74,498	8.7	13.4	1.2	3.8
	R2	20,833	79,897	10.2	11.3	0.8	3.6
	R3	241,103	45,502	6.6	12.9	1.3	2.8
10-TF HAMO-lost	R1	145,226	111,278	5.3	18.3	0.7	1.8
	R2	24,360	72,854	4.7	15.3	0.7	2.8
	R3	–	63,090	–	14.6	–	2.1
Average		59,374	67,268				
Total		1,187,483	1,412,636				

a, 1-Time flush-Feeding; b, 10-Time flush-Feeding; –, not determined.

Supplementary Table 5. MiSeq sequencing summary of 16S rRNA genes in the fractionated DNA isolated from SIP microcosms of paddy soil

DNA Fraction	High Quality Read Number		Type I methanotrophs (%)		Type II methanotrophs (%)	
	$^{12}CH_4$–control	$^{13}CH_4$–labeled	$^{12}CH_4$–control	$^{13}CH_4$–labeled	$^{12}CH_4$–control	$^{13}CH_4$–labeled
14	1	–	–	–	–	–
13	28,020	734	7.8335	–	0.5284	–
12	71	1	–	–	–	–
11	90,131	130,485	4.617	0.584	1.4758	0.141
10	78519	38,532	14.8006	0.4775	0.6701	0.2621
9	122,654	76,217	1.6549	0.8857	0.4692	0.6504
8	25,209	106,827	1.8367	0.7313	0.5316	0.6058
7	106,346	41,508	1.7399	3.4028	0.948	0.9061
6	14,084	18,468	1.2354	56.7468	0.3834	4.0556
5	28	72	–	–	–	–
4	8,432	9,016	1.2935	0.8984	0.3322	9.6939
3	4,271	21,260	1.3349	0.9221	1.1943	3.7967
2	176,983	208,434	2.1025	1.7489	0.1582	1.878
1	9,748	7,886	1.303	0.6596	0.2052	0.5328
Total	664,497	659,440				

–, not determined or not used in further analysis.

Supplementary Table 6. Glycogen metabolism-related gene transcripts detected in HAMO (high-affinity methane oxidation) and non-HAMO soils. Subsystem in MG-RAST (Meta Genome Rapid Annotation using Subsystem Technology) was used for gene function annotation (average e-value ≤ -10, average align length ≥ 30 aa, average % identity ≥ 60). The transcript abundance was normalized based on reads number per million function-known transcripts

Subsystem levels				Abundance	
level 1	level 2	level 3	function	HAMO	Non-HAMO
Carbohydrates	Polysaccharides	Glycogen metabolism	Glycogen phosphorylase (EC 2.4.1.1)	73	42
Carbohydrates	Polysaccharides	Glycogen metabolism	Glucose-1-phosphate adenylyltransferase (EC 2.7.7.27)	67	19
Carbohydrates	Polysaccharides	Glycogen metabolism	1,4-alpha-glucan (glycogen) branching enzyme, GH-13-type (EC 2.4.1.18)	52	23
Carbohydrates	Polysaccharides	Glycogen metabolism	Glycogen synthase, ADP-glucose transglucosylase (EC 2.4.1.21)	38	18
Carbohydrates	Polysaccharides	Glycogen metabolism	Glycogen debranching enzyme (EC 3.2.1.-)	14	11
Carbohydrates	Polysaccharides	Glycogen metabolism	4-alpha-glucanotransferase (amylomaltase) (EC 2.4.1.25)	0	1
